# Peripherally acting anti-CGRP monoclonal antibodies alter cortical gray matter thickness in migraine patients: A prospective cohort study

**DOI:** 10.1016/j.nicl.2023.103531

**Published:** 2023-10-14

**Authors:** Edina Szabo, Sait Ashina, Agustin Melo-Carrillo, Nicolas R. Bolo, David Borsook, Rami Burstein

**Affiliations:** a Department of Anesthesia, Critical Care and Pain Medicine, Beth Israel Deaconess Medical Center, Boston, MA 02215, USA; b Department of Anaesthesiology, Harvard Medical School, Boston, MA 02215, USA; c Comprehensive Headache Center, Beth Israel Deaconess Medical Center, Boston, MA 02215, USA; d Department of Neurology, Beth Israel Deaconess Medical Center, Boston, MA 02215, USA; e Department of Psychiatry, Beth Israel Deaconess Medical Center, Harvard Medical School, Boston, MA 02215, USA; f Department of Psychiatry, Massachusetts General Hospital, Harvard Medical School, Boston, MA 02215, USA; g Department of Radiology, Massachusetts General Hospital, Harvard Medical School, Boston, MA 02215, USA

**Keywords:** Headache, CGRP receptor antagonists, Trigeminal, Prodromes, Pain, Galcanezumab

## Abstract

Migraine is underpinned by central nervous system neuroplastic alterations thought to be caused by the repetitive peripheral afferent barrage the brain receives during the headache phase (cortical hyperexcitability). Calcitonin gene-related peptide monoclonal antibodies (anti-CGRP-mAbs) are highly effective migraine preventative treatments. Their ability to alter brain morphometry in treatment-responders vs. non-responders is not well understood. Our aim was to determine the effects of the anti-CGRP-mAb galcanezumab on cortical thickness after 3-month treatment of patients with high-frequency episodic or chronic migraine. High-resolution magnetic resonance imaging was performed pre- and post-treatment in 36 migraine patients. In this group, 19 patients were classified responders (≥50 % reduction in monthly migraine days) and 17 were considered non-responders (<50 % reduction in monthly migraine days). Following cross-sectional processing to analyze the baseline differences in cortical thickness, two-stage longitudinal processing and symmetrized percent change were conducted to investigate treatment-related brain changes. At baseline, no significant differences were found between the responders and non-responders. After 3-month treatment, decreased cortical thickness (compared to baseline) was observed in the responders in regions of the somatosensory cortex, anterior cingulate cortex, medial frontal cortex, superior frontal gyrus, and supramarginal gyrus. Non-responders demonstrated decreased cortical thickness in the left dorsomedial cortex and superior frontal gyrus. We interpret the cortical thinning seen in the responder group as suggesting that reduction in head pain could lead to changes in neural swelling and dendritic complexity and that such changes reflect the recovery process from maladaptive neural activity. This conclusion is further supported by our recent study showing that 3 months after treatment initiation, the incidence of premonitory symptoms and prodromes that are followed by headache decreases but not the incidence of the premonitory symptoms or prodromes themselves (that is, cortical thinning relates to reductions in the nociceptive signals in the responders). We speculate that a much longer recovery period is required to allow the brain to return to a more ‘normal’ functioning state whereby prodromes and premonitory symptoms no longer occur.

## Highlights

• Anti-CGRP-mAbs change cortical thickness after 3-month treatment.
• Decreased thickness was observed in specific cortical regions in the responders.
• Results could reflect recovery process from the hyperexcitable brain state in migraine.

## 1 Introduction

Migraine is a recurrent pain condition characterized by debilitating unilateral throbbing pain commonly associated with distinct neurological symptoms. In the imaging domain, it is characterized by abnormal brain structure and function (Ashina et al., 2021, Burstein et al., 2015, Schwedt and Dodick, 2009, Sprenger and Borsook, 2012) that can be attributed to disease states (e.g., attack frequency and duration), severity (e.g., pain intensity) and treatment response (Szabo et al., 2021). Structural brain changes are commonly thought to reflect the abnormal neuronal hyperexcitability of the migraine brain (Borsook et al., 2013). This cortical hyperexcitability is considered to play a pivotal role in the enhanced susceptibility of the brain to common migraine triggers and potentially to the ‘generation’ of prodromes (Ashina et al., 2021, Ashina et al., 2023b, Aurora and Wilkinson, 2007, Coppola et al., 2007, Hodkinson et al., 2016, Lang et al., 2004, Moulton et al., 2011, Wilcox et al., 2016).

Increased levels of excitatory (e.g., glutamate) or decreased inhibitory (e.g., GABA) molecules may be affected by the number and density of glial and neuronal cells, and dendritic spine changes in specific cortical regions (including the somatosensory cortex, orbitofrontal cortex, anterior cingulate cortex, insula). These alterations, indicated by increased cortical thickness or cortical thickening, could explain the hyperexcitable brain state in patients with migraine (Borsook et al., 2013). Greater gray matter thickness itself may reflect deficits in pain modulation and hence promote the manifestation of chronic pain. Alternatively, increased cortical thickness could be the consequence of neuro-compensatory processes related to the increased afferent input in migraine (Amin et al., 2021, Borsook et al., 2013, Erpelding et al., 2012, Metz et al., 2009, Teutsch et al., 2008).

One of the main brain regions showing cortical thickening across several studies in migraine is the primary somatosensory cortex (Table 1). Outside the migraine field, this finding has been attributed to the role it plays in sensory aspects of pain perception (recognition of pain location and intensity) (Almeida et al., 2004) and the massive nociceptive input it receives during prolonged pain. In people with migraine, some studies described thickening of the somatosensory cortex in patients with recurrent migraine attacks when compared to patients with lower frequency or no attacks (DaSilva et al., 2007, Kim et al., 2014, Maleki et al., 2012), and others, cortical thinning in older than in younger patients with chronic migraine attacks (Chong et al., 2014, Lai et al., 2020).

**Table 1:** Summary of the cortical thickness alterations in the somatosensory cortex in patients with migraine.

Cortical changes Decreased/Increased cortical thickness	Participants	Notes	References
↓	27 patients with episodic migraine, 32 healthy controls	Patients with migraine showed age-related cortical thinning in regions that did not thin in the pain-free controls.	Chong et al., 2014
↑	12 patients with episodic migraine with aura, 12 patients with episodic migraine without aura, and 12 healthy controls	Thickening of the caudal part of the somatosensory cortex (where the face and the head are represented somatotopically) was found in patients with migraine when compared to controls.	DaSilva et al., 2007
↑	56 patients with episodic migraine and 34 healthy controls	Patients with migraine showed cortical thickening in somatosensory cortex compared to controls.	Kim et al., 2014
↓^a^	30 patients with chronic migraine, 30 healthy controls	Precentral gyrus showed cortical thinning in patients compared to controls.	Lai et al., 2020
↑	10 patients with low-frequency episodic migraine, 10 patients with high-frequency episodic migraine, and 20 healthy controls	Somatosensory cortex showed increased thickness in patients with high-frequency compared to low-frequency migraine.	Maleki et al., 2012

Healthy controls were age- and sex-matched to the patients with migraine.

a Cortical thinning was reported in the precentral gyrus partially overlapping with the somatosensory cortex.

Structural changes may reverse after pain relief with effective treatments. Recently, galcanezumab and other monoclonal antibodies to calcitonin gene-related peptide (anti-CGRP-mAbs) have been introduced into the clinical armamentarium of migraine treatment (Gklinos and Mitsikostas, 2020, Sevivas and Fresco, 2022, Torres-Ferrús et al., 2021). Such studies suggest that around 50 % of patients showed ≥50 % migraine days per month reduction to such treatments (i.e., responder group). In addition, anti-CGRP-mAbs appear to affect different prodromal and accompanying symptoms potentially mediated by the central nervous system (Ashina et al., 2023b, Iannone et al., 2022).

The current longitudinal study aimed at determining whether the prophylactic action of galcanezumab – an anti-CGRP-mAb whose main site of action is thought to reside outside the brain – and its ability to reduce multiple migraine associated symptoms involves restoration of brain structure in regions underlying the generation of migraine attacks and some of its landmark symptoms. Specifically, we assessed the effect of galcanezumab on brain morphometry in cortical regions, before and after 3-month treatment in patients with high-frequency episodic and chronic migraine using MRI. *We hypothesized that changes in cortical thickness would be more pronounced in treatment-responders than treatment non-responders.* Since prior work implicated changes in the primary somatosensory cortex (see Table 1), our hypothesis was that this area would be a primary center for these changes given that one mechanism of galcanezumab is to inhibit or diminish the primary afferent changes from the trigeminal system (Iyengar et al., 2019).

## 2 Materials and methods

The present study was part of a larger project investigating clinical effects of galcanezumab on incidence of headache following occurrence of premonitory symptoms and triggers (Ashina et al., 2023a, Ashina et al., 2023b) and effects on structure and function of cortical areas involved in the genesis of triggers and premonitory symptoms. The study was approved by our local institutional review board (CCI Protocol # 2019P001081) and was conducted according to Good Clinical Practice and the Declaration of Helsinki. Patients provided a written informed consent/assent before inclusion in the study.

### 2.1 Participants

Thirty-six participants (aged between 21 and 63 years old) were included in this prospective, observational open-label cohort study. All patients were recruited at the BIDMC Comprehensive Headache Center, Boston, MA and met the criteria for episodic and chronic migraine according to the third edition of the International Headache Classification of Diseases (IHCD-3; Headache Classification Committee of the International Headache Society IHS (2018)) . Migraine was confirmed by a neurologist and a headache specialist during a clinical interview and after filling out a 4-week electronic diary (e-diary).

Inclusion and exclusion criteria were similar to those reported in our previous studies (Ashina et al., 2023a, Ashina et al., 2023b), with the addition of routine MRI exclusion criteria. Inclusion criteria were ages of 18 and 65 years, previous diagnosis of migraine (with or without aura) in accordance with the IHCD-3 criteria, and onset of migraine at age 50 years or younger. Exclusion criteria were receiving 1 or more migraine preventive treatments, positive pregnancy test, breastfeeding, significant cognitive impairment, psychiatric disorders and/or behavioral problems that could interfere with the study, other significant pain problem (e.g., cancer pain, fibromyalgia, other head or facial pain disorder), severe cardiac disease (e.g., symptomatic coronary artery disease, prior myocardial infarction, congestive heart failure), cerebrovascular disease (e.g., prior stroke or transient ischemic attack, symptomatic carotid artery disease, prior carotid endarterectomy or other vascular neck surgery), report of abnormal electrocardiogram within the last year (e.g., second or third-degree heart block, prolonged QT interval, atrial fibrillation, atrial flutter, history of ventricular tachycardia or ventricular fibrillation, clinically significant premature ventricular contraction), uncontrolled high blood pressure (systolic >160 mm Hg, diastolic >100 mm Hg), known history or suspicion of secondary headache, known history or suspicion of substance abuse or addiction (within the last 5 years), current use of marijuana or has used marijuana (including medical marijuana) or cannabidiol oil within the last 1 year, claustrophobia, and MRI incompatible implants. Other exclusion criteria included current use of opioids for headaches or other body pain, current use of simple analgesics or non-steroidal anti-inflammatory drugs (NSAIDs) ≥15 days per month or triptans, ergots, or combined analgesics ≥10 days per month, receiving nerve blocks (occipital or other) in the head or neck within the last 3 months, receiving onabotulinumtoxinA or anti-CGRP or anti-CGRP-mAb treatment within the last 6 months. Patients were instructed to refrain from initiating or changing type, dosage, or frequency of any medication (e.g., antidepressants, anticonvulsants, beta-adrenergic blocker) during the study.

### 2.2 Study design and data collection

Study participants were scheduled for 3 visits at the Headache Center.

Visit 1: During the first visit, participants received detailed information about the study, gave consent, provided full medical and headache history, and were trained to use a daily electronic headache diary. The headache history was captured in a self-completed questionnaire that included demographics, body mass index (BMI), disease duration, family history, location of headache, frequency of attacks, associated symptoms (symptoms that appear during but not before onset of headache), premonitory symptoms (symptoms that appear before onset of headache, including aura), triggers, list of headache medications, and comorbid conditions. The e-diary was administered in the form of a Research Electronic Data Capture (REDCap) survey using an email link that participants accessed from their personal computer/electronic device. It consisted of a questionnaire that helped determine daily/monthly incidence of headache and migraine, laterality (unilateral, bilateral), pain intensity (0–10 visual analogue scale), and daily occurrence of symptoms necessary to determine whether the headache fulfilled migraine criteria (e.g., nausea, vomiting, throbbing, photophobia, phonophobia, osmophobia). The impact of migraine was assessed with the Migraine Disability Assessment Scale (MIDAS) (Stewart et al., 2000). MIDAS score was calculated by summing the total days missed and days of reduced productivity due to headache from work/school, household work, and nonwork (family, social, leisure) activity over a 3-month period.

Visit 2: During the second visit, which took place after completing the e-diary for 30 days in which no prophylactic treatment was initiated, participants returned to the hospital where they underwent routine pregnancy test at the Clinical Research Center, and MRI scanning at the hospital Imaging Research Center. Following the completion of the MRI scanning, participants returned to the Headache Clinic where they received the first galcanezumab treatment (initiating dose of 240 mg) from the study physician (S.A.) who also trained them on how to self-administer the galcanezumab at home.

Visit 3: During the third visit, which took place 3 months after the second visit, those who treated themselves at home (or returned to the clinic for receiving the 2nd and 3rd injections, 120 mg each) 30 and 60 days after receiving the first injection and completed the e-diary for the entire 120 days (1 month before and 3 months after treatment initiation), returned to the hospital for their second pregnancy test and post-treatment MRI scanning. They were also given the same list of premonitory symptoms and triggers they completed before treatment initiation and were asked to mark those premonitory symptoms that continued to precede the onset of headache and those triggers that continued to cause their headache during the third month of treatment. All post-treatment MRI scans were performed within 3 days from day 90 of the treatment period (i.e., on days 87–90 of the treatment period or days 117–120 of the entire study period). Given the frequency of migraine in many of the participants, we allowed them to be scanned (in visits 2 and 3) when they were headache-free or during mild background headache that was not a migraine (i.e., it did not fulfil any of the migraine criteria, and the pain was so mild that no pain or headache medications were used in the 12 h prior to scanning).

### 2.3 Classification of responders and non-responders

At the end of the study, participants were assigned to one of two groups with respect to their treatment responsivity: treatment responders or treatment non-responders. Responders were those whose monthly migraine days (MMD) decreased by ≥50 % during the 3-month treatment period (calculated based on their daily electronic headache diary during the 30 days before and 90 days after treatment initiation). Non-responders were those whose MMD decreased by <50 % during 3-month treatment period. These definitions are in line with current clinical trial guidelines and recommendations (Detke et al., 2018, Sacco et al., 2020, Stauffer et al., 2018, Tassorelli et al., 2018).

### 2.4 Image acquisition

MRI data were obtained using a 3T MR750 (software version DV26.0) and Signa Premier (DV29.1) scanners (GE Healthcare, Chicago, IL) with a 32-channel NOVA proton head coil. For each participant, T1-weighted structural images were acquired before treatment initiation and again, 3 months after treatment initiation using an accelerated sagittal Inversion Recovery – Fast Spoiled Gradient (IR-SPGR) sequence with number of sagittal slices = 196, slice thickness = 1 mm, no gap, FOV field of view = 256×256 mm, matrix = 256×256, echo time TE = 3.06 ms, inversion time TI = 400 ms, repetition time TR = 7.39 ms, flip angle = 11°, in-plane resolution = 1×1 mm, voxel size = 1×1×1 mm, pixel bandwidth = 244.14 Hz, acceleration factor = 2.

### 2.5 Image processing

Cortical reconstruction and volumetric segmentation of pre- and post-treatment MRI images were performed using FreeSurfer software (version 7.1; https://surfer.nmr.mgh.harvard.edu) (Fischl, 2012). This involves a standard processing pipeline including skull stripping, automated Talairach transformation, segmentation of subcortical gray and white matter, intensity normalization, and gray-white mater boundary tessellation as well as surface deformation. The technical details of these procedures can be found in prior publications (Dale et al., 1999, Dale and Sereno, 1993, Fischl et al., 2004a, Fischl et al., 2004b, Fischl et al., 2002, Fischl et al., 2001, Fischl et al., 1999a, Fischl et al., 1999b, Fischl and Dale, 2000, Han et al., 2006, Jovicich et al., 2006, Reuter et al., 2012, Reuter et al., 2010, Ségonne et al., 2004). These steps were carried out independently for the two timepoints (pre- and post-treatment), and the reconstructed surface models were inspected visually and corrected manually as needed by a rater blind to participant characteristics.

The cross-sectional processing at baseline was followed by the FreeSurfer two-stage longitudinal processing stream to account for the (two-timepoint) repeated-measurement design (Reuter et al., 2012). That is, a “base” (within-subject) unbiased template was created for each participant based on images from both timepoints using a consistent robust inverse registration method (Reuter et al., 2012, Reuter et al., 2010). Cross-sectional images were then re-processed for longitudinal analysis using common information from the base template. The symmetrized percent change was calculated at each vertex from baseline to post-treatment which divides the rate of change by average thickness over the two timepoints. This measure is less dependent on baseline values (compared to other estimates such as percent change) and minimizes the effect of individual variation (Reuter et al., 2012). A smoothing Gaussian Kernel with a full-width half maximum (FWHM) of 15 mm was used. The estimated total intracranial volume (eTIV) was also computed to account for global measures of brain size.

### 2.6 Statistical analysis of demographic and clinical data

No statistical power calculation was conducted prior to the study. The sample size was based on the available data and all analyses were conducted according to the a priori analysis plan. Treatment impact on migraine prodromes and triggers, and its relationship with interictal allodynia in responders and non-responders were previously reported in this patient cohort (Ashina et al., 2023a, 2023b). This study is the primary analysis of the structural MRI study data. Demographic data and clinical measure scores were analyzed with SPSS version 27.0 software (SPSS, Inc., Chicago, IL, USA). Descriptive statistics were calculated (means, standard deviations (*SDs*), median and interquartile range (IQR) for continuous variables; frequencies and percentages for categorical variables) to summarize the sample characteristics. Normality assumptions were tested using Q-Q plots, histograms, and Shapiro-Wilk tests, and the homogeneity of variances was assessed with Levene's test. Mann-Whitney U tests were performed to determine whether responder and non-responder groups differed in age, BMI, clinical features and eTIV. The distribution of categorical variables (sex, aura, handedness, and migraine classification) between groups were analyzed using the Fisher Exact Test.

### 2.7 Cortical thickness and subcortical volume analyses

**Fig. 1:**
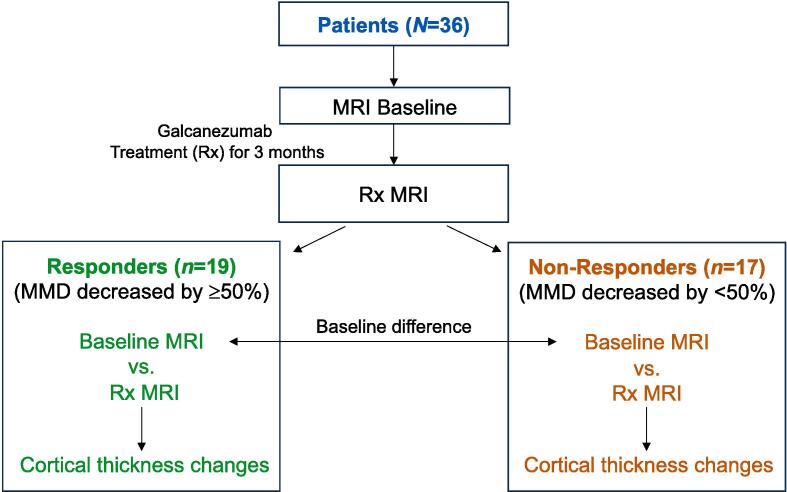
**Flowchart of imaging analysis.** MMD, Monthly migraine days; Rx, Treatment.

Cross-sectional evaluation of cortical thickness differences (to determine whether responder and non-responder groups, and groups with high-frequency episodic or chronic migraine differ at baseline) and longitudinal analysis (to compare cortical thickness at pre- vs. post-treatment) were conducted applying a standard general linear model (GLM) in FreeSurfer (using the tool mri_glmfit) (see Fig. 1 flowchart for an overview of the imagining analysis). Cortical thickness was calculated by measuring the distance from the gray-white matter boundary to the gray-cerebrospinal fluid boundary (Fischl and Dale, 2000). The analyses were performed for the left and right hemispheres separately. For the subcortical structures, volumetric measures at baseline and at 3 months were also tested in GLM for the brainstem and the following nine bilateral structures defined by FreeSurfer (Fischl et al., 2004a): nucleus accumbens, amygdala, caudate, cerebellum, hippocampus, pallidum, putamen, thalamus, and ventral diencephalon. Results for subcortical volumes were corrected for multiple comparisons (adjusted *p* = 0.05/19 = 0.003).

All cortical results were corrected with Monte Carlo simulation, a cluster-wise correction for multiple comparisons (using mri_glmfit-sim, corrected for two hemispheres), and reported with a vertex-wise threshold of *p* < 0.001 and a cluster-wise threshold of *p* < 0.05 (Greve and Fischl, 2018, Hagler et al., 2006). Significant peak coordinates of the observed cortical changes were converted to the Montreal Neurological Institute (MNI152) space. Thickness results were visualized by overlaying the significant cortical areas onto standard pial cortical surfaces.

## 3 Results

### 3.1 Participants' characteristics

The study included 36 patients (29 females, 21–63 years old, *M* age = 34.7, *SD* ± 11.3). Based on the percent reduction in migraine days per month, 19 patients (15 females, *M* age = 33.1, *SD* ± 9.7) were classified as responders (≥50 % reduction in migraine days per month during the first 3 months of treatment) and 17 patients (14 females, *M* age = 36.7, *SD* ± 12.9) were classified as non-responders (<50 % reduction in migraine days per month). Descriptive statistics of the baseline clinical variables for all patients and for the non-responder and responder groups are displayed in Table 2. There was no missing data in the reported dataset. Overall, no significant differences were observed in age, sex, BMI, headache days per month, migraine days per month, years with migraine, headache intensity, impact of headaches (MIDAS score), and aura symptoms between the responder and non-responder groups at baseline. Of note, eTIV did not differ between the groups (responders: 1422487 ± 188649 mm^3^; non-responders: 1416725 ± 154884 mm^3^; *t*(39) = 0.11, *p* = 0.92); therefore it was not entered as a covariate in the group analyses of the structural volume measure differences.

**Table 2:** Baseline demographics, classifications, and disease characteristics of all patients, non-responders and responders.

		All patients	Non-responders	Responders	*p* value
Age					
Mean (SD)		34.7 (11.3)	36.7 (12.9)	33.1 (9.7)	
Median (IQR)			35 (25.5–44)	30 (25–40)	0.52 (Mann-Whitney)

Sex					
N (%)	Female	29 (81 %)	14 (82 %)	15 (79 %)	
	Male	7 (19 %)	3 (18 %)	4 (21 %)	1 (Fisher exact)

Years with migraine					
Mean (SD)		16.3 (9.9)	18 (10.2)	14.9 (9.7)	
Median (IQR)			20 (11.5–25)	13 (7–21)	0.31 (Mann-Whitney)

Migraine days per month					
		15.2 (4.7)	16 (4.8)	14.6 (4.6)	
			15 (12–19.5)	14 (12–15)	0.29 (Mann-Whitney)

Headache intensity					
Mean (SD)		4.2 (1.7)	3.9 (0.9)	4.5 (1.2)	
Median (IQR)			2 (2–2.5)	2 (2–3)	0.13 (Mann-Whitney)

Handedness					
N (%)	Right	29 (80 %)	14 (82 %)	15 (79 %)	
	Left	7 (20 %)	3 (18 %)	4 (21 %)	1 (Fisher exact)

Classification					
N (%)	EM	17 (53 %)	6 (35 %)	11 (58 %)	
	CM	19 (47 %)	11 (65 %)	8 (42 %)	0.20 (Fisher exact)

BMI					
Mean (SD)		25.8 (5.9)	25.2 (6.4)	26.2 (5.6)	
Median (IQR)			24 (21–26.5)	26 (23–27)	0.33 (Mann-Whitney)

MIDAS					
Mean (SD)		34.5 (23.9)	30 (20.6)	38.3 (26.3)	
Median (IQR)			26 (15–43.25)	31 (22–43)	0.24 (Mann-Whitney)

Aura					
N (%)		11 (31 %)	5 (29 %)	6 (31 %)	1 (Fisher exact)

% Decrease in MMD					
Mean (SD)		47.6 (26.6)	24.5 (16.8)	68.4 (13.1)	
Median (IQR)			31.3 (13.5–41.1)	66.7 (58.4–74.3)	<0.0001 (Mann-Whitney)

*Note*. Data are expressed as mean (standard deviation [*SD*]), median (interquartile range [IQR]) and number of participants (N; proportion [%]).

BMI, body mass index; CM, chronic migraine; EM, (high frequency) episodic migraine, MMD, monthly migraine days; MIDAS, Migraine Disability Assessment Scale.

### 3.2 Global cortical thickness changes after 3 months of treatment in all patients

**Fig. 2:**
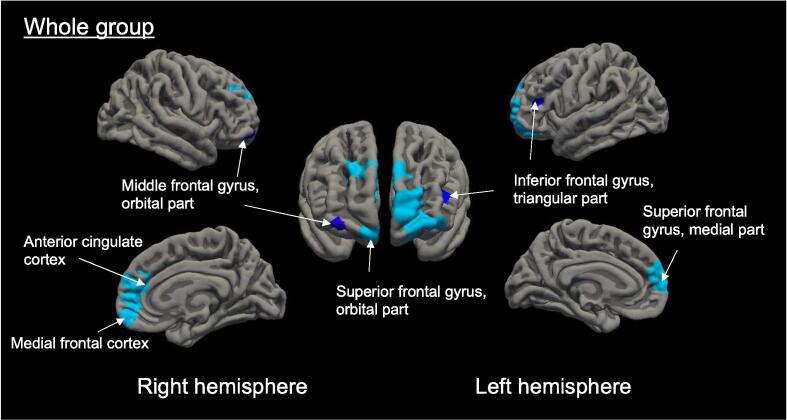
**Cortical thickness changes after 3 months treatment with galcanezumab in all patients (*N* = 36).** Decreased cortical thickness was observed in regions corresponding to the right anterior cingulate cortex, the bilateral orbitofrontal prefrontal cortex, and the triangular part of the left inferior frontal gyrus. Results are multiple comparisons corrected with Monte Carlo simulations (with a cluster-wise threshold of *p* < 0.05). Blue-colored brain regions indicate significant changes (decreased cortical thickness or cortical thinning). Different shades represent distinct clusters on the right and left hemispheres, and light blue indicates cluster-wise *p* < 0.001.

Decreased cortical thickness within the whole group was detected mainly in the bilateral orbitofrontal and right anterior cingulate areas after 3 months of treatment with galcanezumab (Table 3, Fig. 2). No regions showed significantly increased cortical thickness after treatment.

**Table 3:** Clusters showing decreased cortical thickness in all patients with migraine after 3 months of treatment with galcanezumab.

Peak region	Number of vertices	Cluster size (mm^2^)	Peak MNI coordinates x y z			Corrected cluster-wise *p*-value
R Anterior cingulate cortex	3989	2389.08	15	37	22	<0.001
L Superior frontal gyrus, orbital part	3397	2396.11	–22	55	−7	<0.001
L Inferior frontal gyrus, triangular part	546	378.77	−34	37	12	0.003
R Middle frontal gyrus, orbital part	406	304.68	30	56	−11	0.025

*Note.* R, right; L, left; MNI, Montreal Neurological Institute.

Results are corrected for multiple comparisons using the Monte Carlo simulation, with a cluster-wise threshold of *p* < 0.05.

### 3.3 Cortical thickness and subcortical volume differences at baseline and after 3 months of treatment in the responders and non-responders

At baseline, no significant differences were found in cortical thickness and subcortical volume measurements between the responder group and non-responder group, and between the patients with high-frequency episodic and chronic migraine.

**Fig. 3:**
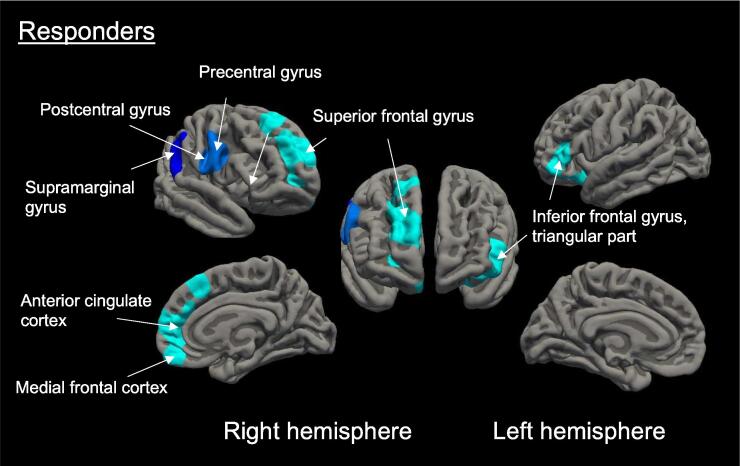
**Cortical thickness changes after 3 months treatment with galcanezumab in responders.** Responders to treatment (*n* = 19) showed decreased cortical thickness in regions corresponding to the right middle and superior frontal gyrus, medial frontal cortex, precentral and postcentral gyrus (somatosensory cortex), anterior cingulate, supramarginal gyrus, and the triangular part of the left inferior frontal gyrus. Results are multiple comparisons corrected with Monte Carlo simulations (with a cluster-wise threshold of *p* < 0.05). Blue-colored brain regions indicate significant cortical thinning. Different shades represent distinct clusters on the right and left hemispheres, and light blue indicates cluster-wise *p* < 0.001.

**Fig. 4:**
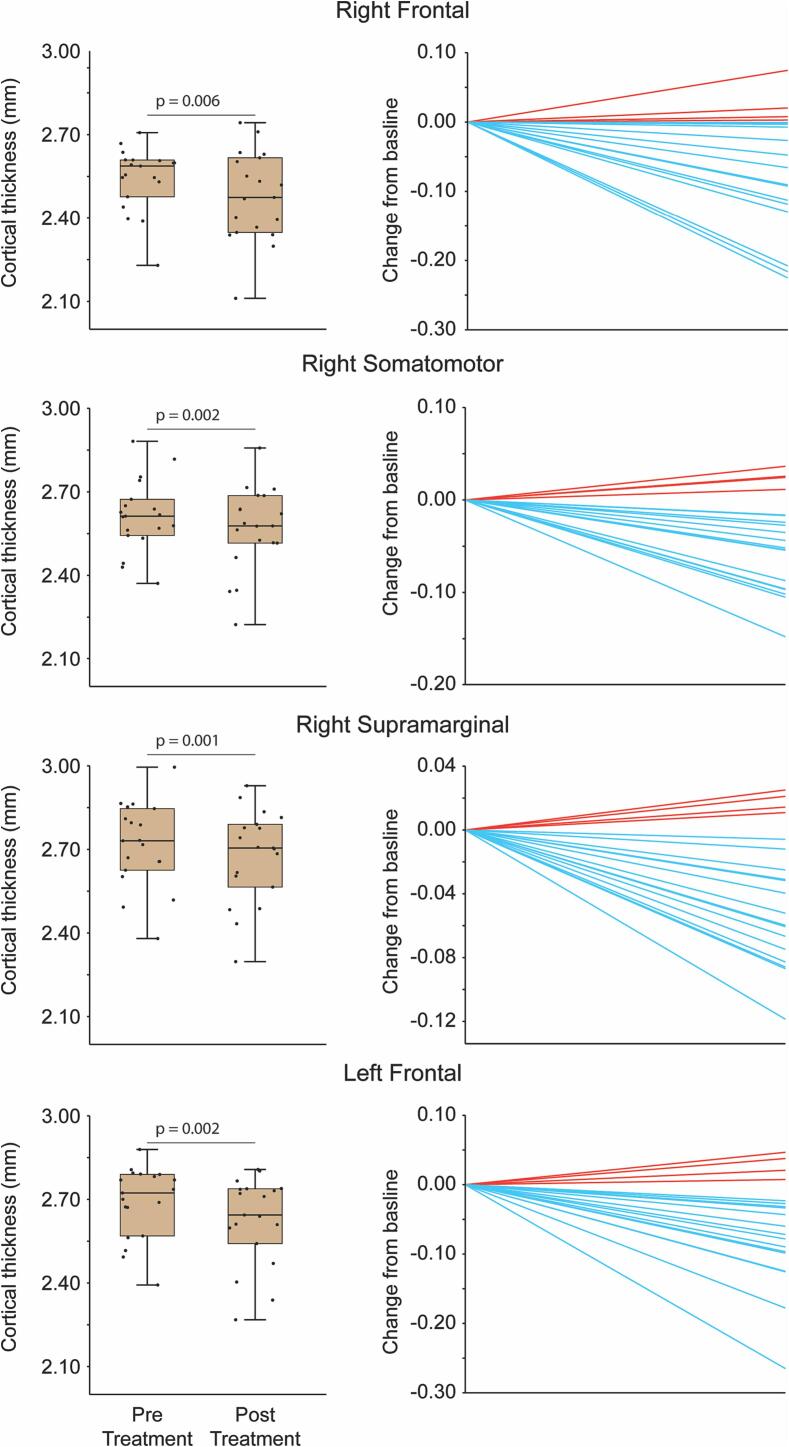
**Quantitative analysis of cortical thickness changes after 3 months treatment with galcanezumab in responders.** Left column – boxplots and scatterplots illustrating average cortical thickness before and after treatment. Note significant decrease in gray matter of the identified cortical regions (Wilcoxon signed ranked test). Right column – cortical thickness change from baseline for each participant. Note that cortical thickness decreased in the vast majority of the responders. Blue lines depict cortical thinning, red lines depict cortical thickening. Median and interquartile range [IQR] values of changes are: right frontal (−0.05[−0.12 to −0.001]), right somatosensory (−0.04 [−0.09 to −0.01]), right supramarginal (−0.04 [−0.07 to −0.006]), left frontal (−0.06 [−0.09 to −0.02]).

**Fig. 5:**
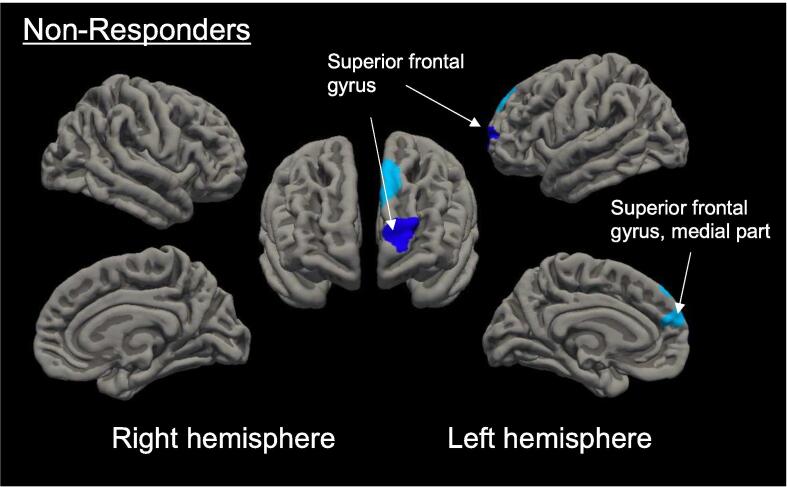
**Cortical thickness changes after 3 months treatment with galcanezumab in non-responders.** Non-responders to treatment (*n* = 17) demonstrated decreased cortical thickness in regions of the left superior frontal gyrus and dorsomedial cortex. Results are multiple comparisons corrected with Monte Carlo simulations (with a cluster-wise threshold of *p* < 0.05). Blue-colored brain regions indicate significant cortical thinning. Different shades represent distinct clusters, and light blue indicates cluster-wise *p* < 0.01.

After 3 months of treatment, in the responder group, surface-based analysis revealed decreased cortical thickness in four clusters, predominantly in the regions of right medial frontal cortex, superior frontal gyrus, precentral and postcentral gyrus (sensorimotor cortex), supramarginal gyrus, anterior cingulate cortex, and left inferior frontal gyrus (triangular part) (Table 4, Fig. 3 and Fig. 4). For visualization purposes, these results are also displayed on the Desikan-Killiany cortical atlas (Desikan et al., 2006) (see Supplementary Fig. 1). In the non-responder group (<50 %), decreased cortical thickness was observed in the left superior frontal and medial superior frontal gyrus (Table 4, Fig. 5). No regions showed a significant increase in cortical thickness after treatment.

**Table 4:** Clusters showing decreased cortical thickness in responders and non-responders after 3 months of treatment with galcanezumab.

Peak region	Number of vertices	Cluster size (mm^2^)	Peak MNI coordinates x y z			Corrected cluster-wise *p*-value
Responders						
R Superior frontal gyrus	7086	4349.22	21	55	24	<0.001
R Precentral gyrus	2542	1183.52	58	2	28	0.012
R Supramarginal gyrus	2151	974.96	60	−40	25	0.038
L Inferior frontal gyrus, triangular part	2007	1275.07	−41	40	3	<0.001

Non-responders						
L Medial superior frontal gyrus	858	482.86	−8	58	25	0.002
L Superior frontal gyrus	456	321.62	−19	62	10	0.015

*Note.* R, right; L, left; MNI, Montreal Neurological Institute.

Results are corrected for multiple comparisons using the Monte Carlo simulation, with a cluster-wise threshold of *p* < 0.05.

None of the subcortical volume changes survived the Bonferroni correction for multiple comparison (*p* < 0.003) in the responders and non-responders. For explorative purposes, the results without the corrections are presented in the Supplementary Material (Supplementary Fig. 2).

## 4 Discussion

In this prospective longitudinal study, we scanned brains of migraine patients before and 3 months after treatment with galcanezumab, an anti-CGRP-mAb approved by the FDA for the prophylactic treatment of migraine. Surface-based analysis was used to evaluate structural brain changes (i.e., repeated measurements of cortical thickness) in areas previously reported to play a role in migraine pathophysiology. We report a widespread reduction of gray matter thickness in the sensorimotor cortex, supramarginal gyrus, prefrontal cortex, and anterior cingulate cortex of migraine patients whose number of monthly migraine days decreased by ≥50 %. In contrast, in patients in which monthly migraine days decreased by <50 %, a decrease in cortical thickness was found only in the left prefrontal cortex. These findings suggest that secondary to the reduction in the number of nociceptive signals that originate in the meninges, brain areas that receive these signals through the many trajectories of the trigeminovascular system and process the overall experience of migraine pain and its unique premonitory symptoms, triggers, and associated symptoms, begin to recover from their hyperexcitable/hyperresponsive state. Mechanistically, it is likely that the decreased cortical thickness reflects changes in dendritic complexity (Borsook et al., 2013) or shrinkage of cortical neurons upon their return to a less active and more normal (or less excitable) excitability level (Akita and Fukuda, 2020). In addition to providing some mechanistic clues and potential links to how galcanezumab, a peripherally acting drug, reduces incidence of headache after occurrence of some premonitory symptoms and ability of some migraine triggers to initiate an attack (as reported in our previous work, Ashina et al., 2023b), this study calls attention to the distinction between site of action and mechanism of action. While galcanezumab’s main site of action is the meninges (i.e., outside the brain) (Noseda et al., 2020), where it neutralizes CGRP, its prophylactic mechanism of action must reside in the brain, where it indirectly reduces neuronal excitability, responsivity, and sensitivity.

### 4.1 Possible mechanism/s of cortical changes in response to treatment with galcanezumab

In patients in which MMD decreased by ≥50 %, we found cortical thinning in multiple cortical areas. While little is known about cellular and molecular mechanisms that determine cortical thickness, existing data strongly correlate cortical thickness increases with greater synaptic density (Schüz and Palm, 1989) and neuronal swelling (Akita and Fukuda, 2020). Data supporting contributions of increased synaptic density include increased dendritic numbers, length, and branching (collectively termed dendritic complexity) in animal models of chronic pain (Metz et al., 2009, Mitrić et al., 2019). Data supporting neuronal and astrocytic swelling include evidence for activity-induced cell and oligodendrocyte swelling in the excitable state (Murphy et al., 2017, Walch et al., 2022, Walch and Fiacco, 2022), during wakefulness (Voldsbekk et al., 2020), as a consequence of reduction in extracellular space and glymphatic flow (Xie et al., 2013), and during epileptic seizures, where activity-induced cell and oligodendrocyte swelling, and increased membrane potential resulted in massive increase in intracellular Cl^−^ that increases osmolyte and water entry into the cells (Akita and Fukuda, 2020). Conversely, normalization of the hyperactive/hyperexcitable state could lead to decreased cell volume, loss of dendritic spines and an associated reduction of excitatory synapses (Borsook et al., 2013, Upadhyay et al., 2010).

In the context of the current study, we propose that the pre-treatment thicker cortices could be explained by the repeated and prolonged migraine attacks which give rise to repeated and prolonged flow of nociceptive signals that through the spinal trigeminal nucleus, brainstem, thalamus and hypothalamus reach the different cortical areas and overwhelm them to the extent that they become hyperactive, hyperexcitable, hyperresponsive and hypersensitive. Conversely, we propose that the thinning of the different cortices in the responders reflects shrinkage of neurons and reduced synaptic density following the reduction in MMD and the consequential reduction in the nociceptive signals they receive overtime. If this scenario is correct, it may be easy to understand the importance of reducing MMD as such reduction allows relevant cortical areas more time to recover (i.e., to reach a lower level of membrane potential which leads to reduced intracellular Cl- and exit of water molecules from the cells).

### 4.2 Specificity to migraine

Cortical thinning was observed in regions involved in higher cognitive functions (including the right medial frontal cortex, superior frontal gyrus, supramarginal gyri, inferior frontal gyrus, anterior cingulate cortex and left inferior frontal gyrus). While the role of these cortical areas in cognitive processes – such as detection of unfavorable outcomes, response errors, response conflict, decision uncertainty, retrieval of “remote” and recent memories, speech production and comprehension (e.g., detecting, discriminating, representing, manipulating, and producing speech sounds), difficulty with finding words or constructing sentences, and at times, the perception of having a brain fog (Alexander and Brown, 2011, Bontempi et al., 1999, Deschamps et al., 2014, du Boisgueheneuc et al., 2006, Euston et al., 2012, Golfinopoulos et al., 2011, Hu et al., 2016, Oberhuber et al., 2016, Peng et al., 2021, Peng et al., 2018, Ridderinkhof et al., 2004, Takashima et al., 2006, Yee et al., 2010), disrupted processing of conflict, affect, pain, and anger (Botvinick et al., 2004, Braem et al., 2017, Fuchs et al., 2014, Gungor and Johansen, 2019, Krug and Carter, 2010, Margulies et al., 2007, Sun et al., 2023), and the short-term perceived stress (Wang et al., 2019, Zhu et al., 2022) – is well established, each constitutes only one component of a large, complex, and multifactorial brain network (involving neurons in many cortical, subcortical and brainstem nuclei) that ‘generates’ some of the functions described above (Arnsten, 2015, Arnsten, 2009, Friedman and Robbins, 2022, Kummer et al., 2020, Kurashige et al., 2019). Given that the impact of treatment on cortical thickness was seen in only few of the many brain areas involved in functions that are commonly disrupted before and during migraine, it must be reasoned that the distribution of nociceptive signals that originate in the meninges during the headache phase of migraine is wide enough to allow these selective areas to receive this information and be affected by it (Burstein et al., 1998, Malick et al., 2001, Malick et al., 2000, Noseda et al., 2011). As to why changes in cortical thickness were not seen in all other areas involved in the production/regulation of cognition, irritability, speech, and stress, it is possible that neuronal and astrocytic end-feet swelling, synaptic density, and dendritic complexity take place in some but not all cortical or subcortical brain areas – a possibility supported by studies showing that the cortical thickness changes seen in the areas identified in the current study were also reported in patients suffering migraine (Magon et al., 2019, Mainero et al., 2011), high level of irritability (Dennis et al., 2019), and short-term perceived stress (Zhu et al., 2022).

### 4.3 Cortical changes in response to treatment

*Somato(motor)sensory cortex:* Regarding the thickness of the precentral and postcentral gyri (somatomotor cortex), it is important to note that the decreased cortical thickness was observed in the lower half of the somatosensory homunculus, where the head and face is somatotopically represented (Kuehn et al., 2017, Moulton et al., 2009). Previous reports of increased cortical thickening in the same area of the primary somatosensory cortex in patients with high-frequency migraine compared to patients with low frequency migraine or healthy controls (DaSilva et al., 2007, Kim et al., 2014, Maleki et al., 2012), and of altered function and functional connectivity in the same somatotopic area of patients experiencing higher migraine frequency and estimated lifetime migraine attacks (Hodkinson et al., 2015, Kökönyei et al., 2021, Maleki et al., 2013, Maleki et al., 2012, Solstrand Dahlberg et al., 2018, Szabó et al., 2019, Youssef et al., 2017) further support the view that any reduction in the flow of nociceptive signals from the meninges to the brain (Burgos-Vega et al., 2015, Levy et al., 2019) is expected to reduce excitability state and improve central sensory processing and sensorimotor integration (Boran et al., 2021, Borsook et al., 2013, Pierelli et al., 2013). Following the same logic, we propose that the thinning of the somatosensory cortex in the responders is achieved through galcanezumab’s ability to diminish the afferent nociceptive barrage associated with a migraine attack and thereby normalize brain excitability. It should be also noted that these changes were more pronounced within the precentral gyrus (containing the primary motor cortex) in the responders. The primary motor cortex has an essential role in pain processing and sensorimotor integration (Borsook, 2007, Chen and Wang, 2022, Mercier and Léonard, 2011, Vittersø et al., 2022), and it has been implicated in the ascending trigemino-thalamo-cortical nociceptive pathway (Burstein et al., 2015, Noseda et al., 2011). Increasing evidence also supports the beneficial effect of modulating the motor system on pain reduction (Goudra et al., 2017). For example, repetitive transcranial magnetic stimulation (rTMS) or direct cortical electrical stimulation of the motor cortex have been found to improve various chronic pain conditions (Ramos-Fresnedo et al., 2022). Additionally, connections between the somatosensory cortex and cortical regions such as the primary motor and prefrontal cortices have been demonstrated anatomically (Bedwell et al., 2015, Gellért et al., 2023, Henry and Catania, 2006) and functionally (Rolls et al., 2022, Zhao et al., 2018). Through these connections, which are thought to define the neural substrate of somatomotor sensory integration, changes in somatosensory cortex functions can explain cortical thinning in prefrontal and motor cortices and potentially contribute to the improvement of the migraine disease (Boran et al., 2021).

*Prefrontal and anterior cingulate cortices*: We have previously summarized the connectivity and involvement of prefrontal regions in pain (Peng et al., 2018). As presented in the results section, several prefrontal brain regions showed evidence of cortical thinning in the responders. These include the superior, medial frontal and inferior frontal cortices. Many of these frontal regions show interconnectivity (Peng et al., 2018). While the superior frontal gyrus is mainly involved in cognitive functions including working memory (du Boisgueheneuc et al., 2006, Yee et al., 2010) and control of impulsivity (Hu et al., 2016), the medial frontal cortex, that includes the polar frontal cortex (Peng et al., 2021, Peng et al., 2018) is functionally associated with integration of information reflecting changes in regions involved in the cognitive mechanisms of pain, emotional control, and pain chronicity (Apkarian et al., 2005, Lorenz et al., 2003, Ong et al., 2019, Qi et al., 2022, Seminowicz and Moayedi, 2017, Wiech et al., 2008). Another important aspect of the current results is the affective processing component, involving changes in the medial prefrontal and anterior cingulate cortices (the medial wall of the frontal lobe), which seem to have a regulatory role with regard to limbic areas in generating emotional responses (Etkin et al., 2011). The anterior cingulate in particular has found to be implicated in the affective processing of pain (Bushnell et al., 2013, Fuchs et al., 2014, Gungor and Johansen, 2019, Krug and Carter, 2010, Margulies et al., 2007, Sun et al., 2023). Together with the prefrontal cortex alterations, these findings in the responders could also suggest changes in the affective and cognitive evaluation and modulation of pain in migraine (Kim et al., 2014, Lev et al., 2010, Ong et al., 2019, Torres-Ferrus et al., 2021).

*Supramarginal region*: Decreased cortical thickness was also found in the supramarginal cortex. This area, especially the right side, is considered to be an association region involved in sensory function (including tactile, sensory-motor integration of speech, coding of time, and proprioceptive processing) (Ben-Shabat et al., 2015, Boran et al., 2021, Hayashi and Ivry, 2020, Liang et al., 2012). Again, this region is implicated in a variety of cognitive functions as well as spatial perception which was found to be altered in migraine patients during their attack (Albrecht et al., 2019, Benedek et al., 2002, Boran et al., 2021, Maleki et al., 2021). The right supramarginal gyrus is also implicated in pain and more recently, it was found to be functionally connected to brain areas involved in affective processing of and empathic response to pain (Bukowski et al., 2020, Zhao et al., 2021), further supporting cortical changes underlying the sensory, cognitive and emotional processing of nociception in the responders.

### 4.4 Treatment implications

In a recent study, we reported that before treatment initiation, incidences of prodromes, triggers and aura that were followed by headache were equally high among responders and non-responders, and that during the 3-month treatment with galcanezumab, incidences of headache following occurrence of some premonitory symptoms, aura and exposure to triggers decreased in the responders (Ashina et al., 2023b). Relevant to the findings of the current study is the distinction between incidence of headache after occurrence of prodromes, aura or exposure to triggers vs. the incidence of prodromes and aura themselves. While the incidence of headache decreased significantly in the responders, the incidence of prodromes and aura did not. Mechanistically, these findings raise the possibility that at the 3-month mark (i.e., 3 months after treatment initiation), migraine attacks were still initiated but their ability to advance attacks from the preictal to ictal phase was blocked by the galcanezumab at the meningeal nociceptors site (Melo-Carrillo et al., 2017b). Along this line, it is reasonable to suggest that the observed cortical thinning was strictly a result of decrease in the magnitude of nociceptive signals that reached the identified cortical areas during the 3-month treatment period. Based on the 3-month data, we also speculate that the incidence of premonitory symptoms and aura themselves may decrease sharply if the brain is given a much longer period of ‘break’ from the pain signals that invade it during the repeated and prolonged migraine attacks.

### 4.5 Site of action (trigeminal ganglion) vs. mechanism of action (brain regions)

In a series of in vivo electrophysiological and anatomical studies, we showed that an anti-CGRP-mAb prevents or reduces the ability of cortical spreading depression to activate thinly myelinated meningeal Ad-fibers and high-threshold trigeminovascular neurons in the spinal trigeminal nucleus (Melo-Carrillo et al., 2017a, Melo-Carrillo et al., 2017b) without crossing the blood brain barrier (Noseda et al., 2020). When considering these data together with previous studies showing no presence of anti-CGRP-mAbs or small molecule CGRP receptor antagonists in any of the brain areas in which we observed thinning (Hargreaves and Olesen, 2019, Hostetler et al., 2013, Johnson et al., 2019, Moore et al., 2020), it must be reasonable to propose that the site of action of these migraine preventative molecules is somewhere along the peripheral component of the trigeminovascular pathway, rather than in the brain. But given that pain perception is generated in the somatosensory cortex and that many of the common migraine triggers (e.g., aura, bright and flickering light, perfume, loud noise) and premonitory symptoms (photophobia, phonophobia, feeling stressed, depressed, anxious, and cognitively compromised) require involvement of other cortical (and subcortical) areas, it is also reasonable to believe that the mechanism by which these drugs prevent the initiation of a migraine attack is through their ability to restore normal excitability, responsivity, and sensitivity of neurons in cortical areas that receive nociceptive signals originating in the meninges during the frequent and prolonged headache phases of migraine. For this class of drugs, we therefore propose that their site of action is peripheral (where the headache begins), whereas their mechanisms of action is central (where most migraine attacks begin). Adapting this proposal, it must be noted however, that we still do not know how a premonitory symptom (such as difficulty forming a sentence or yawning) or a trigger (such as sleeping too little, skipping a meal, or even exposure to bright light) activates the meningeal nociceptors or ends up giving rise to a very distinct pain around the eye.

### 4.6 Laterality effect of cortical thickness changes

In the responders, most of the cortical regions (i.e., somato(motor)sensory, prefrontal, anterior cingulate, and supramarginal areas) showed right-sided brain changes, while left-sided change was observed for the inferior frontal cortex only. In general, right hemispheric dominance has been proposed for attention, emotional processing (of negatively valanced stimuli), withdrawal-associated behaviors (Borod et al., 1998, Davidson, 1995, Killgore and Yurgelun-Todd, 2007, Palomero-Gallagher and Amunts, 2022), and related painful experiences (Duerden and Albanese, 2013). Right-sided activation by affective/cognitive processing of pain has been reported by studies in patients with chronic ongoing neuropathy, cluster headaches and episodic migraine (Hsieh et al., 1996, Hsieh et al., 1995, Szabó et al., 2019). It has been also suggested that, in line with our results, activations in the anterior cingulate (mainly implicated in cognitive control, attention, affective and pain processing) along with the inferior frontal gyrus, medial/superior frontal gyri, and inferior parietal lobule (including the supramarginal cortex) could be part of a right-lateralized attentional system responsible for constant state of alertness to pain (Symonds et al., 2006). Thus, our findings could be interpreted as supporting the idea that the dominance of the right-sided cortical changes in the responders are attributed to the reduction of migraine attacks.

### 4.7 Limitations and future directions

There are a number of limitations: (i) *Evaluation of temporal changes of drug effects*: We only assessed cortical thickness changes after 3 months of treatment, and some brain structures may show dynamic alterations with different attack frequencies. Longer duration treatment (6 or 12 months) could result in further improved long-term (clinical) outcomes and could provide more decisive and conclusive results. (ii) *Sex differences*: Although there was no significant difference in sex between the responder and non-responder groups, biological sex differences could not be assessed because of the female predominance in the sample (which is consistent with the literature: migraine has a female to male gender ratio of approximately 3:1) (Bigal and Lipton, 2009). (iii) *Age distribution*: The age range was broad (from 21 years to over 63 years) and the sample size was too small to provide information regarding efficacy for older and younger participants. (iv) *Migraine patients*: This study investigated high-frequency episodic (8–14 migraine days per month) and chronic migraine patients (15 or more migraine days per month). Even though recent studies suggest that the two subgroups may have similar treatment needs (Jedynak et al., 2021, Quintana et al., 2022, Vernieri et al., 2021), and we did not find baseline differences in cortical thickness between them, research on this topic is still lacking. The use of a threshold of 15 migraine days may not adequately reflect the burden and impact of the disease (Ishii et al., 2021). (v) *Baseline measures*: Although there were no differences in cortical thickness and migraine duration at baseline in treatment-responders vs. non-responders, and we used two-stage longitudinal processing to preserve stable within-subject features across all timepoints, other clinical characteristics could help differentiate them and need to be further explored. (vi) *Non-responders*: We did not determine any processes that may contribute to less responsivity to galcanezumab; however, issues such as inherent hyperexcitability of central trigeminovascular neurons (Ashina et al., 2023a, Ferrari et al., 2015), pain catastrophizing, anxiety, depression, and other psychological processes may provide insights into their decreased responsivity. Alternatively, the duration of treatment may have been too short to allow a more complete detection of structural changes. (vii) *Subcortical changes*: While we evaluated subcortical changes in response to anti-CGRP-mAb, only changes in the pallidum and brainstem were observed (see Supplementary material). Brainstem and striata changes in migraine have been reported (Maleki et al., 2011, Petrusic et al., 2019). Such changes in neural volume may contribute to changes in cortical regions since there are well known between-region connections, for example from the basal ganglia to cortical regions (Nakano, 2000) including from the pallidum to frontal regions (Giarrocco and Averbeck, 2023). (viii) *Group comparison of cortical thickness change:* Difference in cortical thickness changes between responders and non-responders was not analyzed since the current study design was optimized to demonstrate pre- and post-treatment changes in the groups separately.

### 4.8 Conclusion

Taking into consideration (a) previous evidence for CGRP mAbs’ ability to prevent activation of meningeal nociceptors by neutralizing CGRP in the meninges, (b) recently-published evidence for galcanezumab’s ability to decrease incidence of headache following occurrence of premonitory symptoms (including aura) or exposure to triggering events in responders and super-responders (Ashina et al., 2023b), and (c) our current finding of cortical thinning in galcanezumab-responders, we speculate that galcanezumab’s site of action is peripheral (where it blocks activation of selected populations of meningeal nociceptors and reduces the number of pain/nociceptive signals they transfer to the brain), and that its prophylactic mechanism of action is central (where it could potentially allow the hyperexcitable, hypersensitive, and hyperresponsive migraine brain to adapt to a normal level of excitability, sensitivity and responsivity). If such trend (i.e., cortical thinning) is maintained for an entire year of treatment, such effect may be considered as one possible marker of a treatment approach with a potential to be considered disease modifier.

### CRediT authorship contribution statement

**Edina Szabo:** Conceptualization, Methodology, Formal analysis, Software, Investigation, Validation, Visualization, Writing – original draft, Writing – review & editing. **Sait Ashina:** Conceptualization, Methodology, Investigation, Validation, Resources, Data curation, Project administration, Writing – review & editing. **Agustin Melo-Carrillo:** Investigation, Methodology, Formal analysis, Visualization, Writing – review & editing. **Nicolas R. Bolo:** Methodology, Software, Investigation, Validation, Supervision, Data curation, Visualization, Writing – review & editing. **David Borsook:** Conceptualization, Methodology, Investigation, Validation, Resources, Supervision, Data curation, Visualization, Project administration, Writing – original draft, Writing – review & editing. **Rami Burstein:** Conceptualization, Methodology, Investigation, Validation, Resources, Supervision, Data curation, Visualization, Project administration, Funding acquisition, Writing – original draft, Writing – review & editing.

## Declaration of Competing Interest

The authors declare that they have no known competing financial interests or personal relationships that could have appeared to influence the work reported in this paper.

## Data Availability

Data will be made available on request.
