# Supplementary material for: Peripherally acting anti-CGRP monoclonal antibodies alter cortical gray matter thickness in migraine patients: A prospective cohort study
Source: Neuroimage Clin. 2023 Oct 14;40:103531. doi: 10.1016/j.nicl.2023.103531 (PMC10623369; doi:10.1016/j.nicl.2023.103531)
Supplement: Supplementary data 1 [file mmc1.docx]

**Peripherally acting anti-CGRP monoclonal antibodies alter cortical gray matter thickness in migraine patients: A prospective cohort study**

Edina Szabo, Sait Ashina, Agustin Melo-Carrillo, Nicolas R. Bolo, David Borsook, and Rami Burstein

Supplementary Material

**Supplementary Figure 1.** For visualization purposes, cortical thickness changes in the responders are displayed on the Desikan-Killiany cortical atlas after 3 months treatment with galcanezumab. Labels and outlines are based on the Desikan-Killiany cortical parcellation (Desikan et al., 2006). Results are multiple comparisons corrected with Monte Carlo simulations (with a cluster-wise threshold of *p*<0.05). Blue-colored brain regions indicate significant cortical thinning. Different shades represent distinct clusters on the right and left hemispheres, and light blue indicates cluster-wise *p*<0.001.

**Reference:**

Desikan, R.S., Ségonne, F., Fischl, B., Quinn, B.T., Dickerson, B.C., Blacker, D., Buckner, R.L., Dale, A.M., Maguire, R.P., Hyman, B.T., Albert, M.S., Killiany, R.J., 2006. An automated labeling system for subdividing the human cerebral cortex on MRI scans into gyral based regions of interest. Neuroimage 31, 968–980. https://doi.org/10.1016/j.neuroimage.2006.01.021

**Supplementary Figure 2.** **Subcortical volume changes after treatment in the responder and non-responder groups.** Responders to treatment showed decreased volume in the brainstem, whereas non-responders to treatment demonstrated increased volume in the right pallidum. Bar graphs indicate mean and individual subcortical volume values. Error bars represent the standard error of the mean. The results did not survive the multiple comparison correction (*p*<0.003).
* *p*<0.05
